# Integrated Analysis of Human Milk Microbiota With Oligosaccharides and Fatty Acids in the CHILD Cohort

**DOI:** 10.3389/fnut.2019.00058

**Published:** 2019-05-16

**Authors:** Shirin Moossavi, Faisal Atakora, Kozeta Miliku, Shadi Sepehri, Bianca Robertson, Qing Ling Duan, Allan B. Becker, Piushkumar J. Mandhane, Stuart E. Turvey, Theo J. Moraes, Diana L. Lefebvre, Malcolm R. Sears, Padmaja Subbarao, Catherine J. Field, Lars Bode, Ehsan Khafipour, Meghan B. Azad

**Affiliations:** ^1^Department of Medical Microbiology and Infectious Diseases, University of Manitoba, Winnipeg, MB, Canada; ^2^Children's Hospital Research Institute of Manitoba, Winnipeg, MB, Canada; ^3^Developmental Origins of Chronic Diseases in Children Network (DEVOTION), Winnipeg, MB, Canada; ^4^Digestive Oncology Research Center, Digestive Disease Research Institute, Tehran University of Medical Sciences, Tehran, Iran; ^5^Department of Pediatrics and Child Health, University of Manitoba, Winnipeg, MB, Canada; ^6^Department of Pediatrics and Larson-Rosenquist Foundation Mother-Milk-Infant Center of Research Excellence, University of California, San Diego, San Diego, CA, United States; ^7^Department of Biomedical and Molecular Sciences, Queen's University, Kingston, ON, Canada; ^8^School of Computing, Queen's University, Kingston, ON, Canada; ^9^Department of Pediatrics, University of Alberta, Edmonton, AB, Canada; ^10^Department of Pediatrics, University of British Columbia, Vancouver, BC, Canada; ^11^Division of Respiratory Medicine, Department of Pediatrics, Hospital for Sick Children, University of Toronto, Toronto, ON, Canada; ^12^Department of Medicine, McMaster University, Hamilton, ON, Canada; ^13^Department of Physiology, University of Toronto, Toronto, ON, Canada; ^14^Department of Agricultural Food, and Nutritional Science, University of Alberta, Edmonton, AB, Canada; ^15^Department of Animal Science, University of Manitoba, Winnipeg, MB, Canada

**Keywords:** *Bifidobacterium*, breastmilk, microbiota, human milk oligosaccharide, fatty acids, mode of breastfeeding, CHILD cohort

## Abstract

**Background:** Human milk contains many bioactive components that are typically studied in isolation, including bacteria. We performed an integrated analysis of human milk oligosaccharides and fatty acids to explore their associations with milk microbiota.

**Methods:** We studied a sub-sample of 393 mothers in the CHILD birth cohort. Milk was collected at 3–4 months postpartum. Microbiota was analyzed by 16S rRNA gene V4 sequencing. Oligosaccharides and fatty acids were analyzed by rapid high-throughput high performance and gas liquid chromatography, respectively. Dimension reduction was performed with principal component analysis for oligosaccharides and fatty acids. Center log-ratio transformation was applied to all three components. Associations between components were assessed using Spearman rank correlation, network visualization, multivariable linear regression, redundancy analysis, and structural equation modeling. *P*-values were adjusted for multiple comparisons. Key covariates were considered, including fucosyltransferase-2 (FUT2) secretor status of mother and infant, method of feeding (direct breastfeeding or pumped breast milk), and maternal fish oil supplement use.

**Results:** Overall, correlations were strongest between milk components of the same type. For example, FUT2-dependent HMOs were positively correlated with each other, and *Staphylococcus* was negatively correlated with other core taxa. Some associations were also observed between components of different types. Using redundancy analysis and structural equation modeling, the overall milk fatty acid profile was significantly associated with milk microbiota composition. In addition, some individual fatty acids [22:6n3 (docosahexaenoic acid), 22:5n3, 20:5n3, 17:0, 18:0] and oligosaccharides (fucosyl-lacto-N-hexaose, lacto-N-hexaose, lacto-N-fucopentaose I) were associated with microbiota α diversity, while others (C18:0, 3′-sialyllactose, disialyl-lacto-N-tetraose) were associated with overall microbiota composition. Only a few significant associations between individual HMOs and microbiota were observed; notably, among mothers using breast pumps*, Bifidobacterium* prevalence was associated with lower abundances of disialyl-lacto-N-hexaose. Additionally, among non-secretor mothers, *Staphylococcus* was positively correlated with sialylated HMOs.

**Conclusion:** Using multiple approaches to integrate and analyse milk microbiota, oligosaccharides, and fatty acids, we observed several associations between different milk components and microbiota, some of which were modified by secretor status and/or breastfeeding practices. Additional research is needed to further validate and mechanistically characterize these associations and determine their relevance to infant gut and respiratory microbiota development and health.

## Introduction

Milk microbiota refers to the microbial community in the milk that the infant receives through breastfeeding. Although intramammary colonization has not been definitively proven (1), several culture-dependent and -independent studies have confirmed the presence of bacteria as well as fungi in human milk (2, 3). Milk serves as a niche for these microbes, and has likely evolved to select and enrich beneficial bacteria for delivery to the infant. Compared to the growing body of evidence identifying modifiable extrinsic factors shaping the milk microbiota (4, 5), the influence of the intrinsic milk environment and its major components is relatively unexplored. We previously modeled the milk environment as an unobservable variable using summary measures (first principal component axes) of human milk oligosaccharides (HMOs) and fatty acids (4). Although we did not find significant associations with the overall milk microbiota composition using these summary metrics, it remains possible that interactions occur between individual bacteria, HMOs, and fatty acids.

HMOs constitute 20% of milk carbohydrates (6). Most studies to-date have focused on HMOs and the infant gut microbiota, especially *Bifidobacterium* and *Bacteroides* (7–10). However, it is less clear whether milk microbiota in general or milk *Bifidobacterium* specifically could also utilize HMOs. A few observational studies have detected positive correlations between total HMO concentration and milk bacteria including *Bifidobacterium* spp. and *Staphylococcus* (11–13). On the other hand, some individual HMOs including lacto-N-fucopentaose (LNFP) I, 2′-fucosyllactose (2′FL), 3′-sialyllactose (3′SL), 6′-sialyllactose (6′SL), and lacto-N-tetraose (LNT) were negatively correlated with Actinobacteria (including bifidobacteria), *Staphylococcus*, and other taxa in milk. Some HMOs have also been positively correlated with potential pathogens such as *Enterobacter/Klebsiella* (13–15). In a small metagenomic study of human milk, HMO metabolism genes were not detected, which was attributed to the absence of *Bifidobacterium* spp. (16). However, other reports observed differences in the prevalence of *Bifidobacterium* according to the maternal secretor status (17), which is a major determinant of HMO synthesis. Notably, none of these studies accounted for the mode of breastfeeding (directly at the breast or pumped and bottled) which was a key determinant of milk microbiota composition in our previous study (4). Overall, these divergent results from relatively small studies highlight the need to examine HMOs and milk microbiota in larger studies while accounting for secretor status, mode of breastfeeding and other potential confounding factors.

Milk lipids are the main source of energy for the infant and primarily consist of triglycerides (6), which are fatty acids bound to glycerol. While the relative amounts of some milk fatty acids are fairly consistent across studies, geographies and individual women (18), others such as docosahexaenoic acid (DHA) are strongly influenced by maternal diet (19). *In vitro* studies have shown that polyunsaturated fatty acids (PUFAs) can have inhibitory effects on bacteria, including *Lactobacillus*, in a dose-dependent manner (20) while 12:0, cis-9/trans-11 and trans-10/cis-12 conjugated linoleic acid (CLA) can inhibit growth of *Staphylococcus aureus in vitro* (21). PUFAs and saturated fatty acids (SFAs) have also been shown to influence the microbiota composition and diversity in the gut (22–24), but their impact on milk microbiota has not been widely studied. Some correlations have been observed between milk fatty acids and milk microbiota (14). Specifically, negative correlations were reported for milk monounsaturated fatty acids (MUFAs) and PUFAs with Proteobacteria, *Streptococcus, Acinetobacter, Lactobacillus*, and *Bifidobacterium* while SFAs were positively correlated with *Pseudomonas* (25). However, these correlations have not been examined while controlling for maternal fish oil supplement use, which modifies milk n3-PUFA concentrations (26) and might also influence microbiota (27).

**Figure 1:**
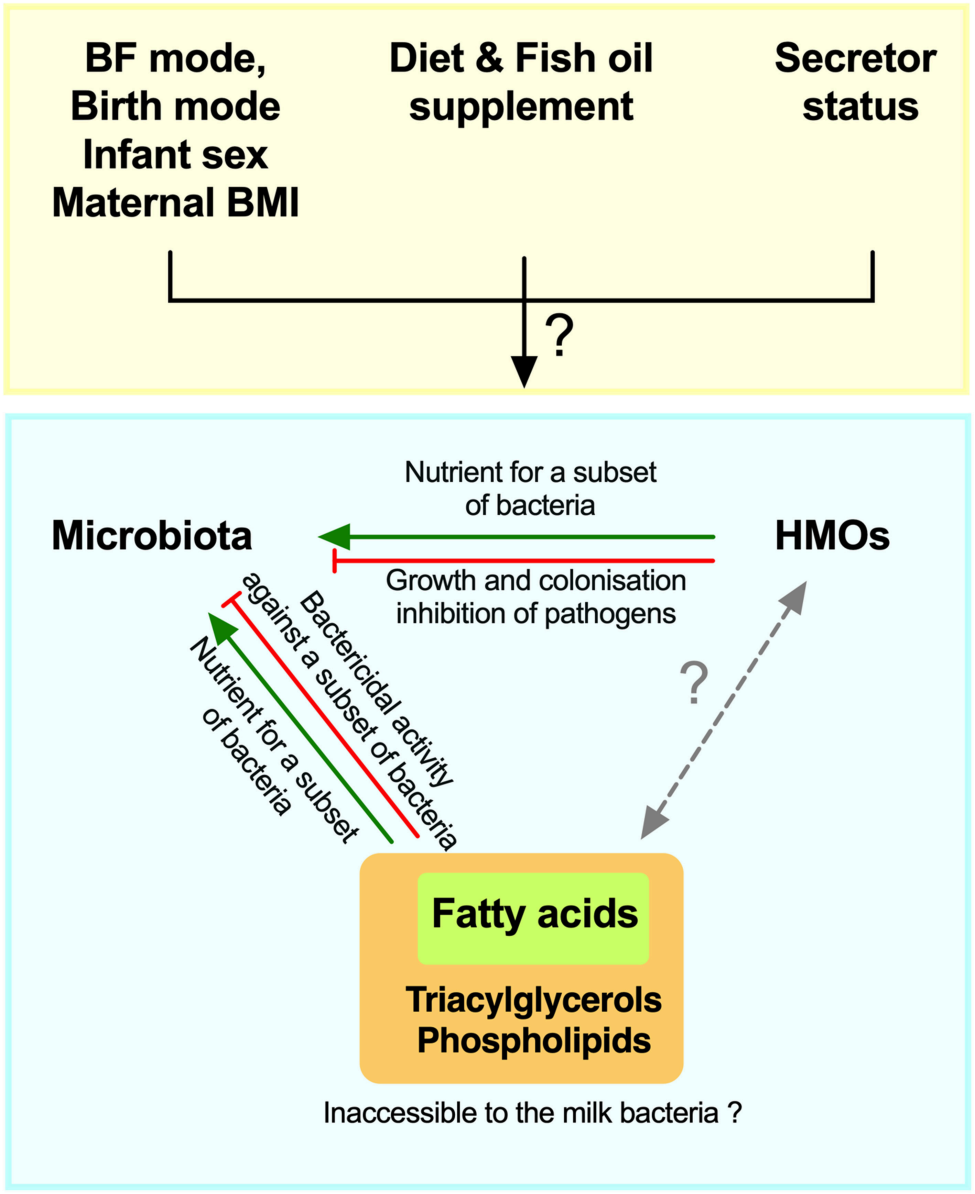
Schematic of hypothesized integrative associations of human milk oligosaccharides (HMOs) and fatty acids, with milk microbiota, and factors that could modify these associations. BF, breastfeeding; BMI, body mass index.

Given the suggestive evidence noted above and the potential roles of HMOs and fatty acids in promoting or inhibiting bacterial growth in experimental settings (23, 28), we hypothesized that these different milk components could shape the milk microbiota composition and could be influenced by maternal secretor status, fish oil supplement use, and breastfeeding practices. Therefore, the objective of this study was to perform an integrative analysis of milk microbiota with HMOs and fatty acids while taking into account key factors that impact these milk components (Figure 1).

## Materials and Methods

### Study Design

We studied a representative subset of 393 mothers participating in the Canadian Healthy Infant Longitudinal Development (CHILD) birth cohort (29). Women with singleton pregnancies were enrolled between 2008 and 2012 and remained eligible if they delivered a healthy infant >35 weeks gestation (*n* = 3,455). We selected a representative subset of mother-infant dyads with available milk samples (*N* = 432), ensuring equal representation across the four study sites, excluding dyads missing key maternal (e.g., diet, BMI) or infant (1y clinical) data, and then randomly selecting among the rest (4). For the current study, samples with sufficient depth of sequencing for microbiota analysis (*N* = 393) were included (Supplementary Methods). All participants gave written informed consent in accordance with the Declaration of Helsinki. The protocol was approved by the Human Research Ethics Boards at McMaster University, the Hospital for Sick Children, and the Universities of Manitoba, Alberta, and British Columbia.

### Milk Sample Collection

Each mother provided one sample of milk at 3–4 months postpartum [mean (SD) 17 (5) weeks postpartum] in a sterile milk container provided by the CHILD study. To control for differences in the milk composition of fore- and hindmilk (30) as well as the diurnal variation (31), a mix of foremilk and hindmilk from multiple feeds during a 24-h period was collected. Hand expression was recommended, but pumping was also acceptable. The sample was not collected aseptically. Samples were refrigerated at home for up to 24 h before being collected and processed by study staff (32). Samples were stored at −80°C until analysis.

### DNA Extraction and Microbiota Analysis

Milk microbiota was assessed by 16SrRNA gene sequencing of V4 hypervariable region with modified F515/R806 primers (33) on a MiSeq platform (Illumina, San Diego, CA, USA) and processed using dada2 (34) as previously described (4) (Supplementary Methods). Potential reagent contaminants were identified and excluded using decontam package (35). Demultiplexed sequencing data was deposited into the Sequence Read Archive (SRA) of NCBI and can be accessed via accession number SRP153543.

### HMO Analysis

Human milk oligosaccharides (HMOs) were previously measured in the same milk samples by high performance liquid chromatography (36). The following 19 HMOs were detected on the basis of retention time comparison with commercial standard oligosaccharides and mass spectrometry analysis: 2′FL, 3-fucosyllactose (3FL), 3′SL, 6′SL, LNT, lacto-N-neotetraose (LNnT), LNFPI, LNFPII, LNFPIII, sialyl-LNT (LST) b, LSTc, difucosyl-LNT (DFLNT), disialyl-LNT (DSLNT), fucosyl-lacto-N-hexaose (FLNH), difucosyl-lacto-N-hexaose (DFLNH), fucosyl-disialyl-lacto-N-hexaose (FDSLNH), and disialyl-lacto-N-hexaose (DSLNH), difucosyllactose (DFLac), and lacto-N-hexaose (LNH) and were quantified based on response factors. These 19 HMOs typically account for >90% of total HMO content; their concentrations were summed to estimate total HMO concentration. The relative abundance of each HMO was calculated. The amount of HMO-bound fucose (Fuc) and HMO-bound sialic acid (Sia) were calculated. HMOs were also grouped according to common structural elements as previously described (37) (Table S1). Maternal secretor status was defined by the presence or near absence of 2′FL or LNFP I. HMOs were studied as both absolute concentrations (μg/mL) and relative proportions.

### Fatty Acids Analysis

Fatty acids were also previously analyzed by gas liquid chromatography at University of Alberta as previously described (38). The following 29 fatty acids were detected: capric acid (10:0), lauric acid (12:0), myristic acid (14:0), physeteric acid (14:1n9), pentadecylic acid (15:0), palmitic acid (16:0), 7-hexadecenoic acid (16:1n9), margaric acid (17:0), stearic acid (18:0), trans vaccenic acid (TVA, 18:1t11), oleic acid (18:1n9), cis-vaccenic acid (18:1c11), linoleic acid (LA, 18:2n6), arachidic acid (20:0), γ-linoleic acid (GLA, 18:3n6), α-linolenic acid (ALA, 18:3n3), eicosadienoic acid (20:2n6), dihomo-γ-linolenic acid (DHGLA, 20:3n6), arachidonic acid (AA, 20:4n6), conjugated linoleic acid (CLA, family of isomers of linoleic acid), eicosapentanoic acid (EPA, 20:5n3), nervonic acid (24:1n9), docosahexaenoic acid (DHA, 22:6n3), lignoceric acid (24:0), osbond Acid (22:5n6), docosapentaenoic acid (DPA, 22:5n3), eicosatetraenoic acid (20:4n3), adrenic acid (C22:4n6). Fatty acids were grouped into SFAs, MUFAs, and PUFAs. Additionally, they were classified as synthesized *de novo* in the mammary gland, or acquired from the diet, liver synthesis, or body fat storage. PUFAs were also divided into n-3 and n-6 series (Table S1).

### Covariates

The mode of breastmilk feeding was reported for breastfed infants at the time of breastmilk collection and classified as “direct breastfeeding only” (feeding at the breast only, with no feeding of pumped milk), or “some pumped milk” (at least one serving of pumped milk in the past 2 weeks) (39). Maternal age, infant sex, birth weight, gestational age, method of birth, and parity were documented from hospital records. Method of birth was categorized as normal vaginal delivery, emergency, or elective Cesarean section. Maternal ethnicity and fish oil supplement use during pregnancy was self-reported by standardized questionnaire.

### Infant Secretor Status

Infant DNA was isolated from peripheral blood samples. Genotyping of single nucleotide polymorphisms (SNPs) was performed using the HumanCoreExome BeadChip (Illumina, San Diego, CA, USA) comprising >240 K tagSNPs and >240 K exome-focused markers. A stringent process of quality control at infant and SNP levels was applied using PLINK v.1.9 (http://pngu.mgh.harvard.edu/purcell/plink/href) (40). Samples were excluded if demonstrated low genotype call rates (missingness >0.10), discrepancies with reported sex, were ethnic outliers based on PCA, or were related as determined by identity by descent. SNPs were excluded with missingness >0.10, had significant departures from Hardy-Weinberg equilibrium (*p* <10^−6^), or had a minor allele frequency <0.01. IMPUTE2 software (41) was used to impute genotypes based on the 1000 Genomes Project reference panel (42), which resulted in >23,000,000 single nucleotide variants of high quality (>0.7). Using this imputed dataset, infant secretor status was defined based on FUT2 rs601338 or rs1047781 SNPs.

### Statistical Analysis

Data analysis was conducted in R (43). α diversity was assessed by the observed OTUs (richness) and inverse Simpson index (diversity). To control for the compositional nature of the data, microbiota, fatty acids, and HMO relative abundances were center log-ratio transformed following zero-replacement (44, 45). After this transformation, correlations of taxa relative abundances with fatty acids and HMOs were compared at phylum, family, and genus levels by Spearman rank correlation. *P*-values were corrected with Benjamini–Hochberg's false discovery rate (FDR) method. Analyses of HMOs and fatty acids were stratified by maternal secretor status and fish oil supplement use, respectively. Additionally, partial correlations were assessed using Spearman rank correlation and visualized as networks using qgraph package (46). The association of HMOs and fatty acids composition profiles with milk microbiota composition was assessed by redundancy analysis (RDA) with 1,000 permutations using the vegan package (47). Structural equation modeling (SEM) was performed using the lavaan package (48). Model fit was assessed by χ^2^ test, the comparative fix index (CFI), and the standardized root mean residuals (SRMR). Non-significant χ^2^ test, CFI ≥ 0.9, RMSEA < 0.05, and SRMR < 0.08 were considered as indications of good model fit (49). Association of HMOs Z normalized relative abundances with *Bifidobacterium* presence was assessed using logistic regression stratified by mode of breastfeeding and adjusted for maternal secretor status. Covariate selections for different statistical tests were informed by our previous report of the association of maternal, infant, and early life factors with milk microbiota composition (4). *P*-values of less than 0.05 were considered significant.

## Results

The majority of our representative subset of 393 mothers were Caucasian (74%) and the mean ± SD maternal age was 33 ± 4 years. About half (54%) were primiparous and a quarter (24%) delivered by Cesarean section. The mean ± SD lactation stage at sample collection was 17 ± 5 weeks postpartum and 58% of mothers were providing some pumped milk to their infant. Seventy-two percent of mothers were secretors and 21% used fish oil (Table S2).

**Figure 2:**
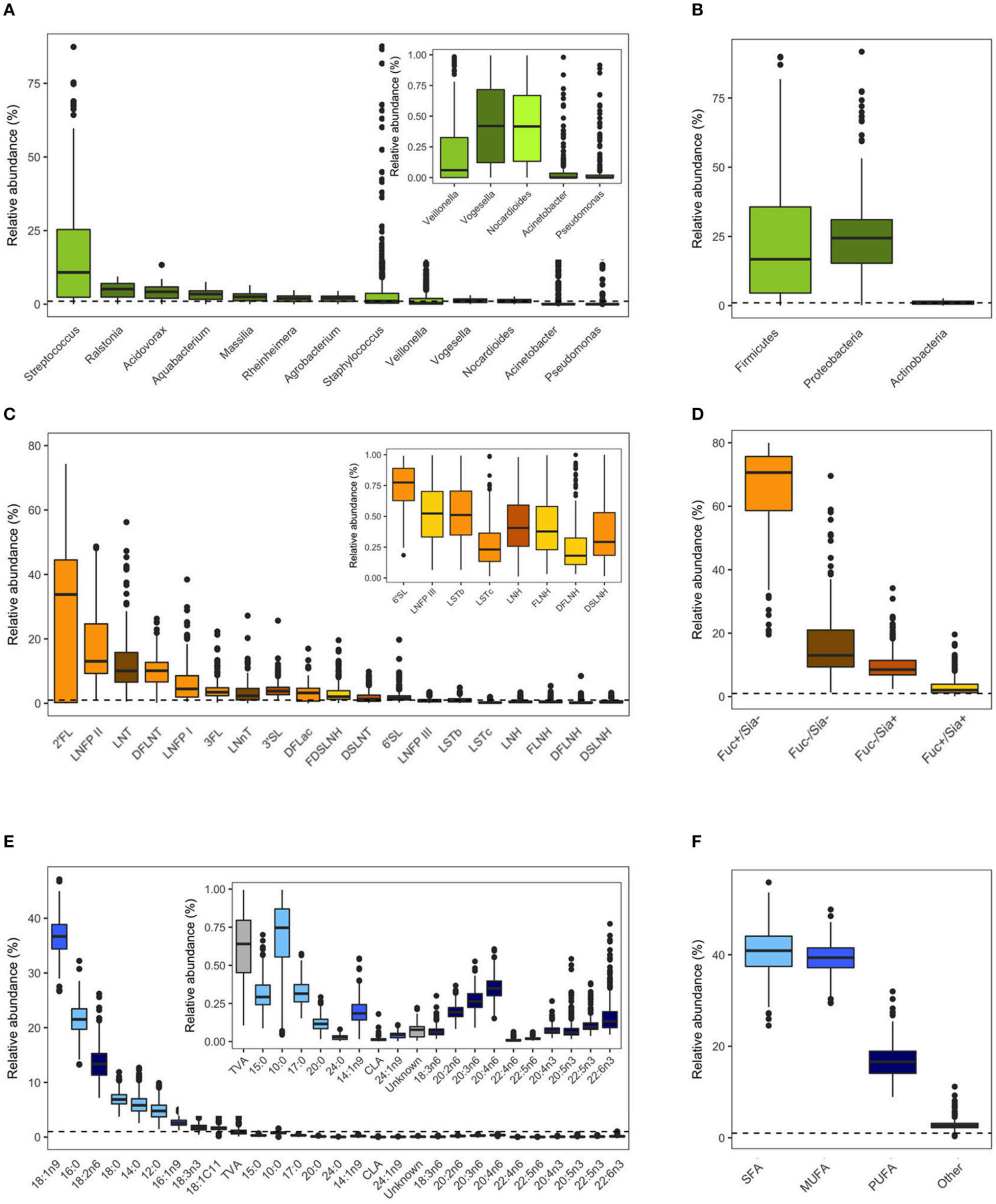
Milk microbiota, human milk oligosaccharides (HMOs), and fatty acids relative abundances. **(A)** Abundant bacteria genera (>0.01% mean relative abundance), **(B)** aggregated relative abundances at the phylum level, **(C)** HMOs, **(D)** HMO groups based on fucosylation and sialyation, **(E)** fatty acids, **(F)** fatty acids categories. Dashed horizontal lines mark 1% relative abundance; components with means <1% are shown in figure insets. Figures are adapted from results originally reported in Moossavi et al. (4), Azad et al. (36), and Miliku et al. (50).

Milk microbiota and HMO profiles in the CHILD cohort have been previously described (4, 36, 50). Overall, *Streptococcus, Ralstonia*, and *Acidovorax* were the most abundant genera (Figure 2A) and Proteobacteria (mean 67%) and Firmicutes (mean 26%) were the most abundant phyla (Figure 2B). 2′FL (among secretor mothers), LNFP II, and LNT were the most abundant HMOs (Figure 2C) and fucosylated/non-sialylated was the most abundant HMO group (Figure 2D). Fatty acids oleic acid (18:1n9), palmitic acid (16:0), and linoleic acid (18:2n6) were the most abundant while the majority had very low relative abundances (<1%) (Figure 2E). SFAs (41%) and MUFAs (39%) were the most abundant fatty acids followed by PUFAs (17%) (Figure 2F).

### Correlations Are Stronger and More Frequent Within Than Between Different Types of Milk Components

**Figure 3:**
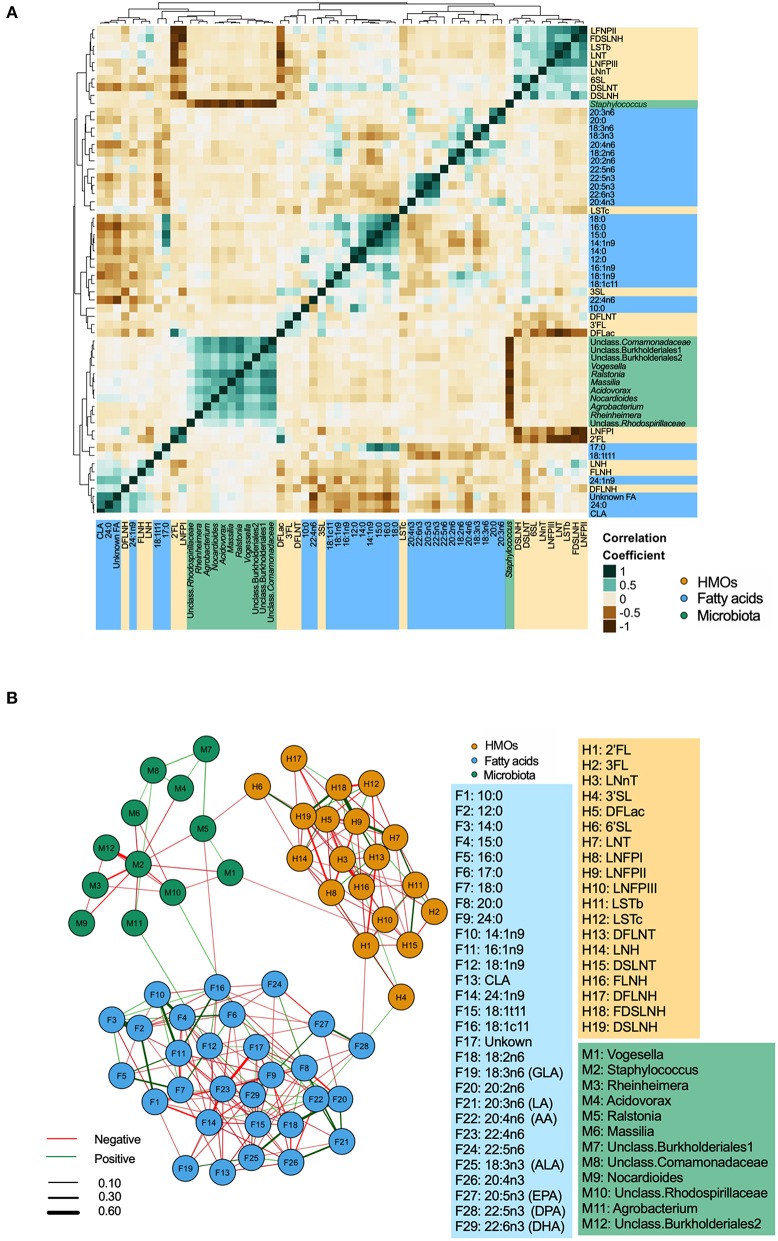
Inter and intra-correlations of milk microbiota, human milk oligosaccharides (HMOs), and fatty acids. **(A)** Clustering heatmap based on Spearman rank correlation, **(B)** Network visualization based on partial Spearman rank correlations. Line color represents the direction of correlation: green (positive), red (negative). Line width is proportional to the correlation coefficient.

Integrated correlations of different components were visualized in a heatmap. The strongest correlations were observed within each group (Figure 3A). For example, *Staphylococcus* was strongly negatively correlated with nearly every other core taxa (*r*-values <-0.60, *p* < 0.05). Strong positive correlations were also found between LNFP II/FDSLNH, C12:0/C14:0, 22:4n6/24:1n9 (all *r* > 0.4, *p* < 0.05). Next, to isolate and visualize the most significant associations, we assessed pairwise partial correlations between members of milk microbiota, HMOs, and fatty acids using Spearman correlation. Correlations with *p*-value of < 0.05 after Benjamini–Hochberg's correction were visualized as a network (Figure 3B). Consistent with the conventional heatmap, the network shows that the number and strength of intra-group correlations were higher than inter-group correlations, which could be partly attributed to the compositional nature of all three components. Partial correlations between core microbiota and fatty acids included Agrobacterium/18:1n9 (positive), and *Ralstonia*/18:1c11 (negative). Correlations between microbiota and HMOs included *Vogesella*/DSLNT (negative) and *Ralstonia*/6'SL (negative) (Figure 3B).

### Some HMOs and Fatty Acids Are Associated With Milk Microbiota α Diversity and Overall Composition

**Figure 4:**
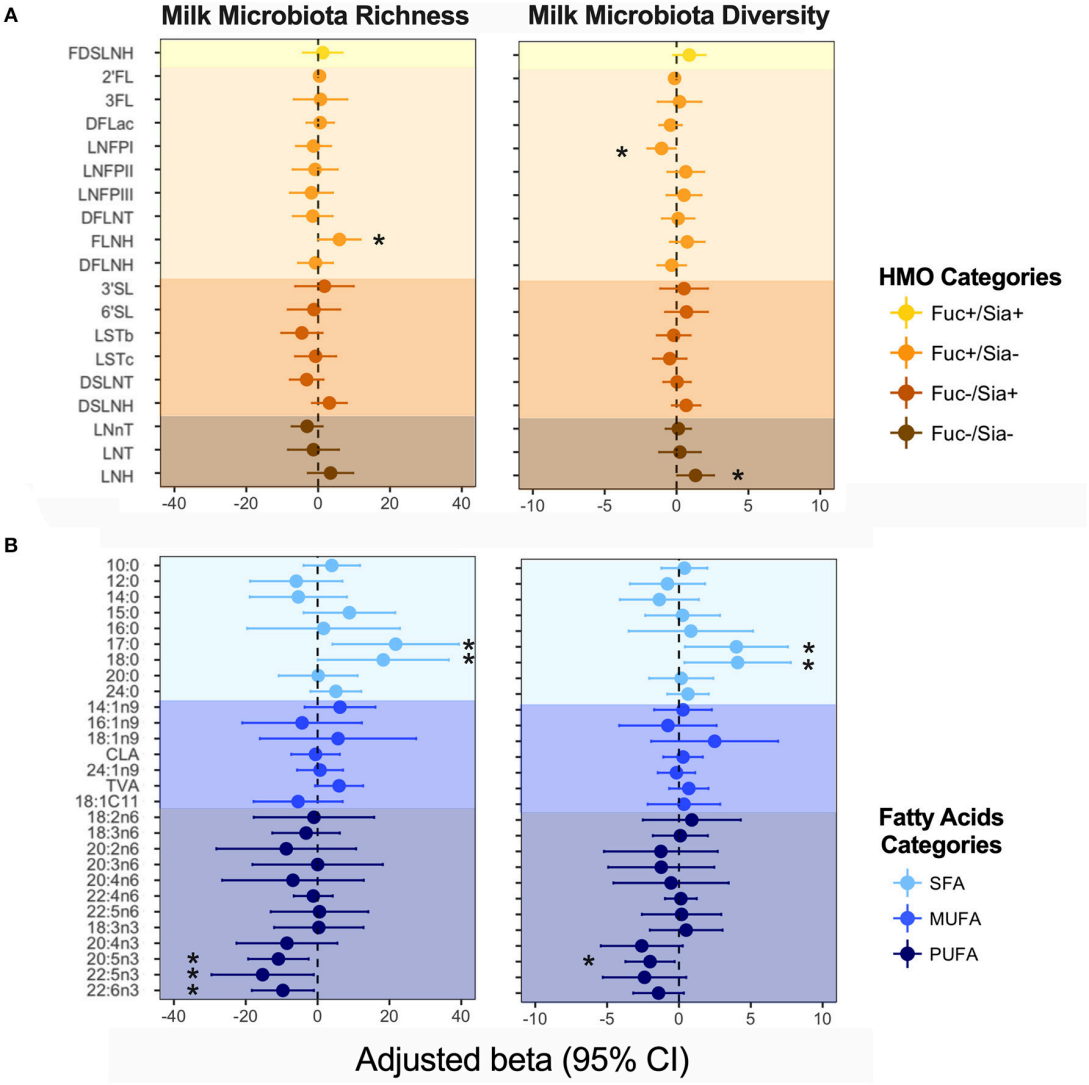
Association of **(A)** human milk oligosaccharides (HMOs) and **(B)** fatty acids with milk microbiota richness (observed ASVs) and diversity (inverse Simpson index). Adjusted for mode of breastfeeding, infant sex, parity, and birth mode. Fatty acids and HMOs are colored according to their categories. ^*^denotes significant associations (*p* < 0.05).

We previously identified mode of breastfeeding, infant sex, parity, and birth mode to be associated with microbiota richness and diversity, but found no association between milk microbiota diversity and the first principal component axes (PC1) for either HMOs or fatty acids (4). However, when examining individual HMOs and fatty acids (expressed as relative proportions) in the current analysis, some associations were observed. Controlling for the above-mentioned covariates, FLNH was associated with microbiota richness [β = 5.90, 95%CI (0.04–11.8) per SD increase in FLNH, *p* = 0.04) while LNH (β = 1.32, 95%CI (0.04–2.60), *p* = 0.04] and LNFP I (β = −1.04, 95%CI (−2.03 to −0.05), *p* = 0.03] were associated with microbiota diversity (Figure 4A). These opposite effects of different HMOs could explain the apparent lack of association of the overall HMO profile and microbiota diversity. Other HMOs were not associated with milk microbiota richness and diversity, and we did not detect any significant patterns of association when grouping HMOs according to fucosylation and sialyation (Figure 4A) or other common structural elements (37) (not shown).

Fatty acids DHA, DPA, EPA (all n3-PUFAs) were negatively associated with milk microbiota richness [e.g., DHA: β = −9.67, 95%CI (−18.30 to −1.031), *p* = 0.028], while SFAs 17:0 and 18:0 were positively associated with these metrics (Figure 4B). We did not detect any associations for MUFAs (Figure 4B), nor for fatty acids grouped based on their source/origin (i.e., synthesized *de novo* in the mammary gland, or acquired from the diet, liver synthesis, or body fat storage) (Figure S1).

Using redundancy analysis, overall HMO and fatty acid compositions were not associated with the overall composition of the milk microbiota (4.82% variation explained, *p* = 0.30 for HMOs and 7.60%, *p* = 0.13 for fatty acids; Table 1), as previously observed (4). Consistent with this finding, milk microbiota diversity and taxonomic clusters were not associated with the overall composition of HMOs or fatty acids (Figure S2). However, a few significant associations were observed when examining individual HMOs and fatty acids. Specifically, HMO 3′SL (0.58%, *p* = 0.01) and DSLNT (0.45%, *p* = 0.04) and fatty acid 18:0 (0.55%, *p* = 0.01) were significantly associated with the overall composition of the milk microbiota (Table 1).

**Table 1:** Association of microbiota with human milk oligosaccharides (HMOs) and fatty acids.

**Independent profile or component**	**Dependent profile**					
	**Microbiota**		**HMOs**		**Fatty acids**	
	**%^a^**	***p***	**%^a^**	***p***	**%^a^**	***p***
**HMO compositional profile**	4.82		–		11.50	^***^
3FL	0.19		–		0.40	~
LNnT	0.36		–		0.46	^*^
3SL	0.58	^**^	–		0.78	^**^
DFLac	0.23		–		0.65	^**^
6SL	0.27		–		1.44	^***^
LNT	0.19		–		0.46	^*^
LNFPIII	0.17		–		0.92	^***^
LNH	0.28		–		1.10	^***^
DSLNT	0.45	^*^	–		3.13	^***^
FDSLNH	0.17		–		0.37	~
**Milk fatty acid profile**	7.60		10.50	^**^	–	
12:0	0.24		0.21	^*^	–	
16:0	0.18		0.33	^**^	–	
16:1n9	0.30		0.15	^*^	–	
18:0	0.55	^*^	0.03		–	
18:2n6	0.41	~	0.02		–	
20:3n6	0.30		0.12	~	–	
24:0	0.39	~	0.07		–	
24:1n9	0.38	~	0.08		–	
22:5n3	0.41	~	0.04		–	

a *Values reflect the percentage of variation explained using redundancy analysis*.

*Only those with significant association with at least one component are reported. ~p <0.10*,

* *p < 0.05*,

** *p < 0.01*,

*** *p < 0.001*.

### Some HMOs and Fatty Acids Are Weakly Associated With Specific Milk Bacteria Relative Abundances

Next, we assessed the correlation of individual HMOs and fatty acids with milk microbiota at phylum, family, and genus taxonomic levels (all taxa with >0.01% mean relative abundance). Only a few weak (*r* < |0.15|) associations between HMOs and microbiota were identified. These associations were mostly between DFLac, type 1 HMOs (consisting of LNT, LNFP I, LNFP II, LSTb, and DSLNT), and non-fucosylated/non-sialylated HMOs (LNnT, LNT, LNH) with Bacteroidetes and *Prevotellaceae* (Figure S3). When stratified by maternal secretor status, only sialylated/non-fucosylated HMOs remained significantly correlated with *Prevotella* (*r* = 0.22, *p* = 0.028). We did not detect any significant associations with HMOs expressed as absolute quantities (not shown).

Similarly, we identified a few weak correlations (*r* <|0.20|) between fatty acids and milk bacteria including negative correlations between C18:1c11/Actinobacteria and CLA/*Porphyromonadaceae*, and positive correlations between C20:4n6/Bacteroidetes, C22:5n3/Fusobacteria, and 22:4n6/*Prevotellaceae*. No correlations with any of the fatty acid categories (SFAs, MUFAs, or PUFAs) were observed (not shown).

**Figure 5:**
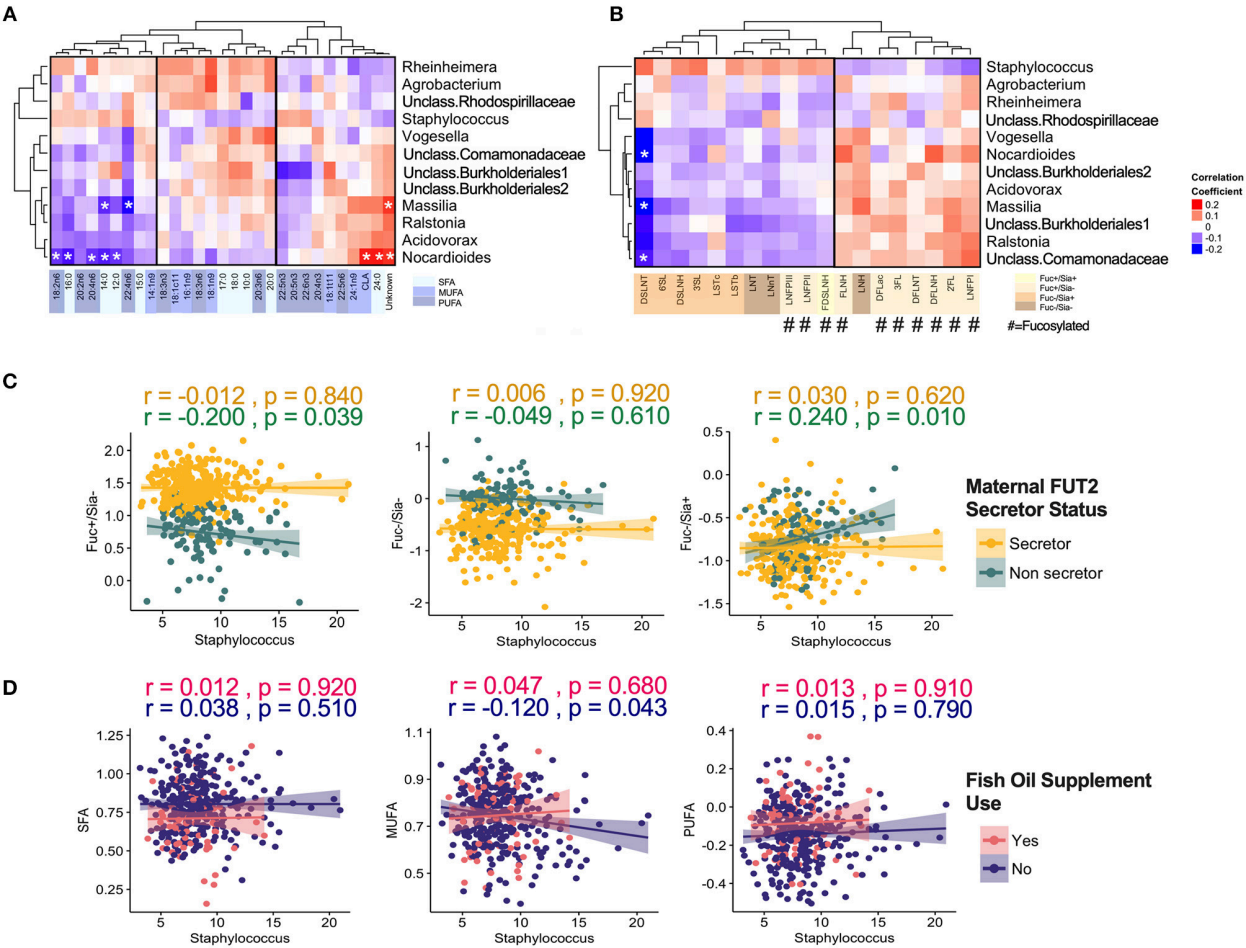
Correlation of core milk microbiota (>1% relative abundance) with milk components. **(A)** Fatty acids, **(B)** human milk oligosaccharides (HMOs). Correlation of core *Staphylococcus* ASV with **(C)** HMOs stratified by maternal secretor status and **(D)** Fatty acids groups stratified by maternal fish oil supplement.

When focusing on the core milk microbiota [amplicon sequence variants (ASVs) present in at least 95% of samples with more than 1% mean relative abundance], some additional patterns emerged. Overall, core microbiota was weakly correlated with fatty acids and HMOs (Figure 5). *Nocardioides* was significantly associated with several fatty acids including 12:0, 14:0, 16:0, 24:0 (SFAs, correlations with *r* > |0.20| and *p* < 0.05) (Figure 5A). The strongest individual correlations were observed for DSLNT with *Nocardioides, Massilia, and unclassified Comamonadaceae* (negative correlations with *r* <−0.23 and *p* < 0.05) (Figure 5B). In general, the directions of correlation of core ASVs with HMOs were opposite for fucosylated vs. sialylated oligosaccharides. These correlations were directionally consistent across most taxa, with the notable exception of *Staphylococcus:* while most ASVs were positively correlated with fucosylated HMOs and negatively correlated with sialylated HMOs, these relationships were inversed for *Staphylococcus* (Figure 5B). Interestingly, when stratified by secretor status, these associations were only observed in non-secretor mothers (Figure 5C). Additionally, MUFAs were significantly associated with Staphylococcus in the absence of fish oil supplementation (Figure 5D).

### Maternal and Infant Secretor Status Are Not Strongly Associated With Overall Milk Microbiota Composition

**Figure 6:**
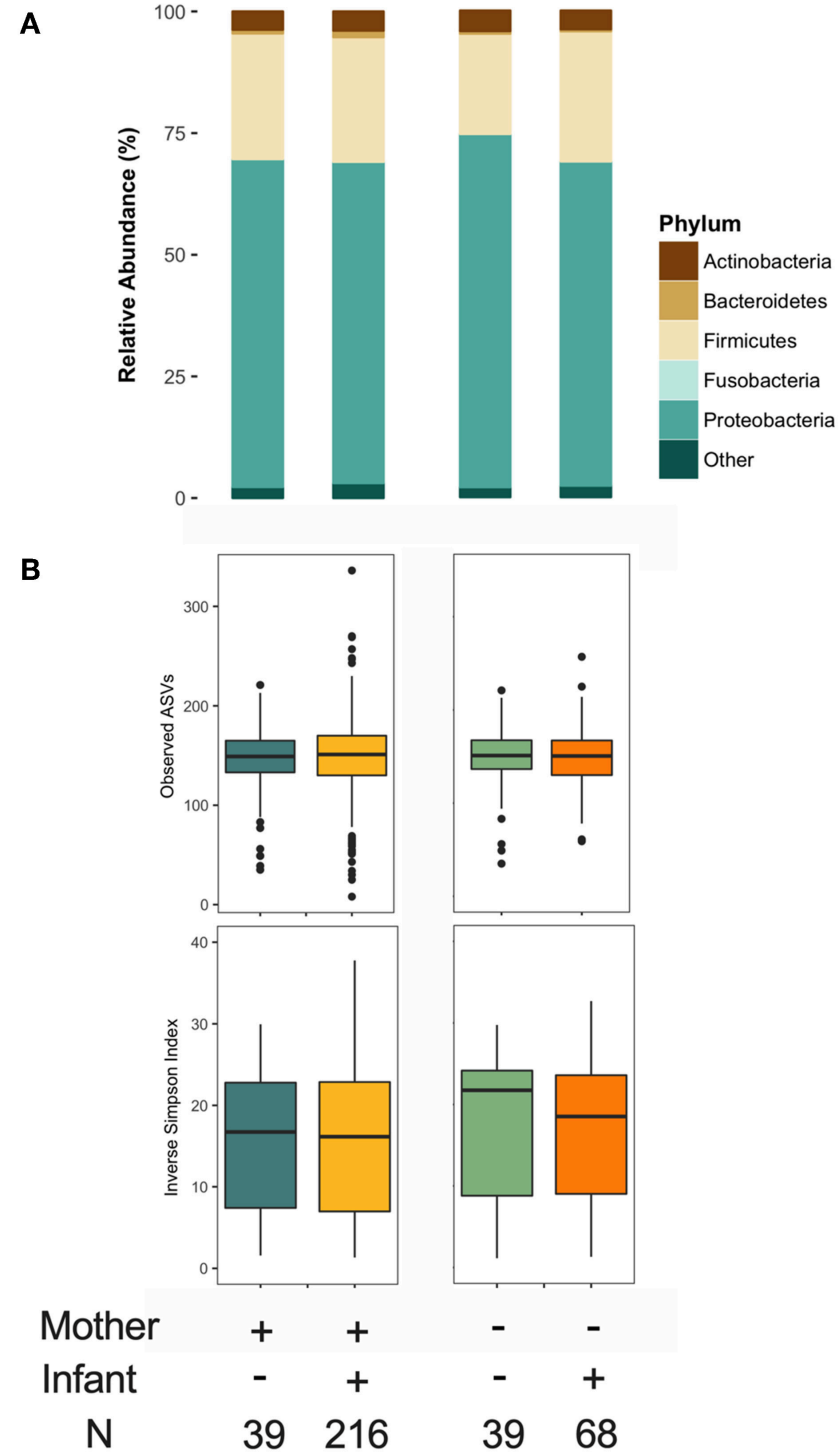
Association of maternal and infant secretor status with milk microbiota. **(A)** Taxonomy at phylum level and **(B)** α diversity.

As previously reported (36), maternal FUT2-secretor status was a clear determinant of HMO composition (Figure S4). We also assessed infant secretor status because this genotypic trait could influence the infant oral microbiota, which could potentially inoculate the mother's milk. We did not identify major differences in the milk microbiota structure or α diversity based on maternal or infant secretor status (Figure 6). However, Bacteroidetes relative abundance was significantly higher in milk produced by secretor mothers (β = 2.15, 95% CI 0.56–3.73 for secretor vs. non-secretor mothers, *p* < 0.01, independent of mode of breastfeeding and other relevant covariates) (Figure 6A). This difference was driven by enrichment of *Prevotella* (0.5 ± 1.7 vs. 0.2 ± 0.7, *p* < 0.05). There was also a trend in the association of infant secretor status with milk Actinobacteria diversity (β = 0.37, 95% CI −0.031 to 0.775, *p* = 0.07 for secretor vs. non-secretor infants). Maternal and infant secretor statuses were not associated with *Bifidobacterium* relative abundance or prevalence (not shown), or milk microbiota β diversity (Figure S4A). Total HMO concentration was not associated with milk microbiota structure (not shown).

### HMOs Are Associated With Milk *Bifidobacterium* Prevalence and Abundance

**Figure 7:**
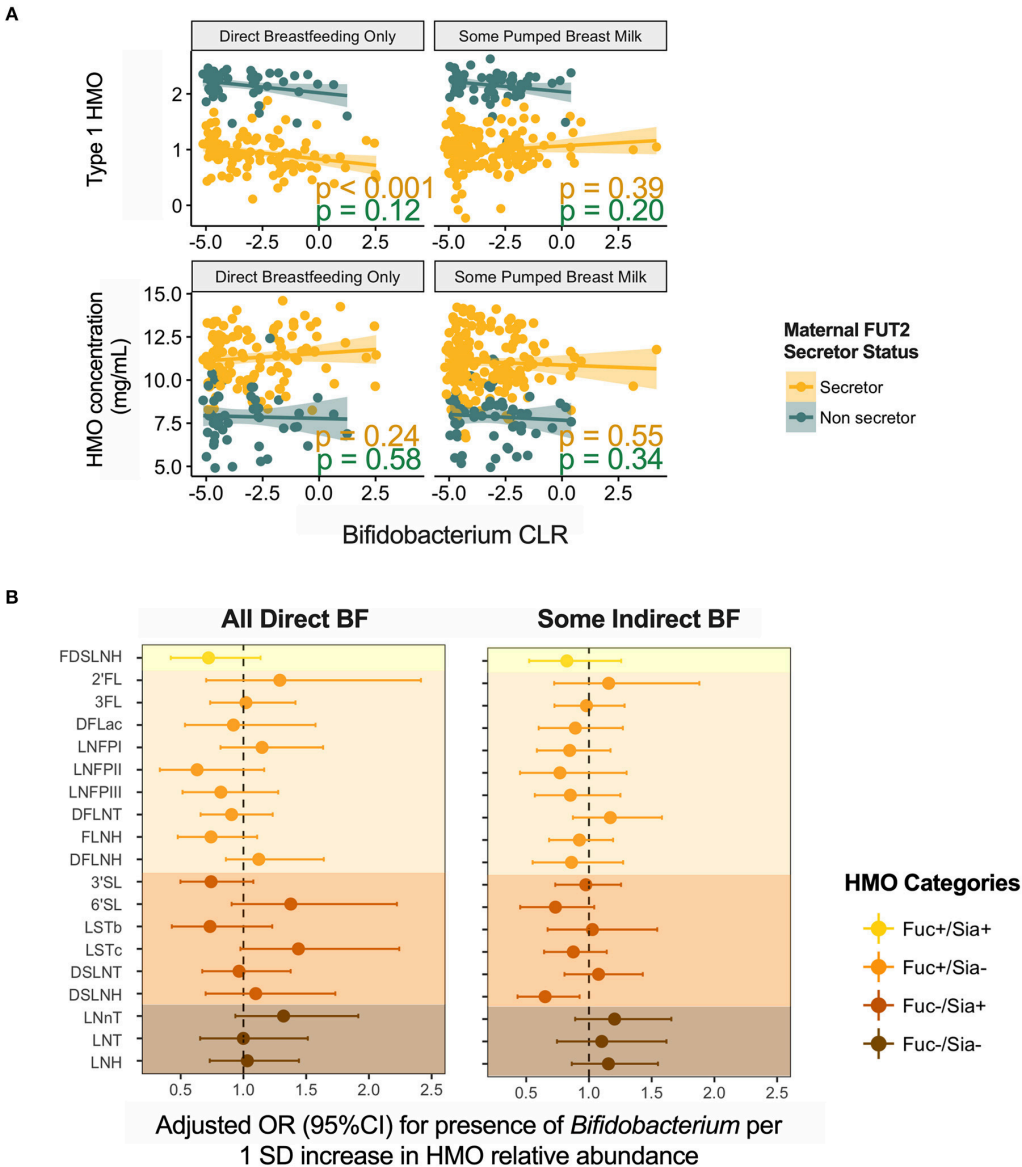
Association of *Bifidobacterium* **(A)** relative abundance and **(B)** prevalence with human milk oligosaccharides. BF, breastfeeding.

Given the established ability of bifidobacteria to consume HMOs, we specifically explored the association of HMOs and bifidobacteria in milk. *Bifidobacterium* was present in 154 (39%) samples with mean relative abundance of 0.25 ± 0.98%. Overall, we did not observe significant associations for total or individual HMO concentrations or relative abundances (not shown); however, patterns emerged after stratification on feeding method. We undertook stratification because we have previously shown that *Bifidobacterium* is more prevalent in milk microbiota of mothers feeding exclusively at the breast (4). Among these mothers, *Bifidobacterium* relative abundance was negatively associated with type 1 HMOs (*r* = −0.29, *p* < 0.001) (Figure 7A). Among mothers providing some pumped breast milk, *Bifidobacterium* was less likely to be present in milk with higher levels of DSLNH (OR = 0.65, 95%CI 0.43–0.93, *p* = 0.03 per 1 SD increase in DSLNH, Figure 7B).

### Milk Fatty Acid Profile Is Associated With Milk Microbiota Composition When Controlling for Relevant Confounding Factors

**Figure 8:**
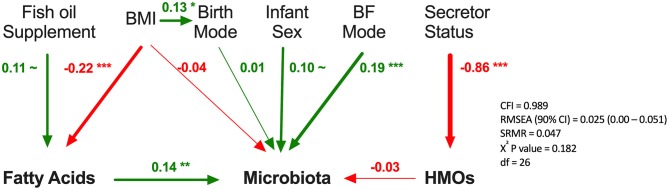
Structural equation modeling of the association of human milk oligosaccharides (HMOs) and fatty acids with microbiota. Standardized β coefficients are reported. BF, breastfeeding; BMI, maternal body mass index; CFI, comparative fix index; CI, confidence interval; RSMEA, root mean square error of approximation; SRMR, standardized root mean residuals; ~*p* < 0.1, ^*^p < 0.05, ^**^p < 0.01, ^***^p < 0.001, Green (positive), red (negative).

Finally, we used SEM to assess the associations between milk components using summary statistics (first principal component axes; PC1) while controlling for key covariates (Figure 8). Fatty acids PC1 was significantly associated with milk microbiota composition (PC1) when controlling for fish oil supplement use (a key dietary source of n3-PUFA) and maternal BMI (a key factor influencing fatty acids originating from liver synthesis or body storage), maternal secretor status, mode of breastfeeding, infant sex, and birth mode (β = 0.14, *p* < 0.01). HMO PC1 was not significantly associated with milk microbiota composition in this model. Thus, consistent with results from redundancy analysis, the SEM results suggest that fatty acids, more so than HMOs, are associated with milk microbiota composition while controlling for relevant factors (Figure 4).

## Discussion

To our knowledge, this is the first integrative analysis of human milk microbiota with HMOs and milk fatty acids. We applied multiple statistical approaches to combine and simultaneously analyse the association of these milk components with milk microbiota while controlling for important covariates reflecting maternal genetics, diet, and breastfeeding practices. Overall, the correlations between milk components within each group were stronger and more frequent than correlations between components from different groups. Still, a few weak associations were observed between individual HMOs or fatty acids and microbial taxa, including *Bifidobacterium*. Interestingly, some of these associations were modified by maternal secretor status, fish oil supplementation, or method of breastfeeding. In structural equation models controlling for these and other key factors influencing milk composition, the overall fatty acid profile (but not the overall HMO profile) remained directly associated with milk microbiota composition.

Our study contributes new evidence on the potential interactions between HMOs and milk bacteria. Some bacterial genera are known to either utilize HMOs (e.g., *Bifidobacterium, Bacteroides*, and *Lactobacillus*) or benefit from their growth-promoting effect without direct utilization (e.g., *Staphylococcus*) (12, 28, 51). We identified correlations between certain HMOs and Bacteroidetes, *Bifidobacterium*, and *Staphylococcus*. The correlations were relatively weak and occurred in both positive and negative directions, in agreement with the different HMO consumption characteristics of these bacteria (12, 28). However, these correlations were different from previous small-scale q-PCR-based (11, 17) and sequencing-based studies (13, 14, 17). Notably, Bacteroidetes relative abundance was higher in milk from secretor mothers suggesting preferential utilization of α-1,2 HMOs. Lack of major shifts in the mature milk microbiota by maternal secretor status was in accordance with previous studies (15, 17). Interestingly, while nearly all core milk microbiota demonstrated specific and inverse correlation patterns with fucosylated and sialylated HMOs (which could indicate utilization or inhibition), these relationships were inversed for *Staphylococcus*. Although *Staphylococcus* does not utilize HMOs, it could still be influenced by the presence of HMOs in the microenvironment (12). Finally, HMO 3′SL and DSLNT (α2-3-sialylated HMOs) were significantly associated with the overall composition of the milk microbiota (52), suggesting that some HMOs might indeed shape milk microbiota. Interestingly, some *Bifidobacterium* spp. found in milk have sialidase enzymes with activity on α2-3 linkages, enabling them to release free sialic acid from these particular HMOs (52). However, whether these HMOs are metabolized by the majority of milk bacteria is not known. Given the large abundance of lactose as a more accessible carbon source in milk, it is also not known if HMOs are a major source of energy for milk bacteria.

We specifically investigated *Bifidobacterium* because this species is predominant in the gut of breastfed infants, yet the source of this bacterium is unclear. Interestingly, only 39% of human milk samples in our study contained *Bifidobacterium*, which is curious in light of the current consensus that *Bifidobacterium* is vertically transferred via breastmilk to “seed” the infant gut. Notably, our samples were collected at 3–4 months postpartum, so it remains possible that bifidobacteria are more ubiquitously present earlier in lactation. Previous studies examining strains of *Bifidobacterium* in infant gut, mother's milk, and maternal gut have concluded that they could be transferred from mother to infant through milk (53, 54). If this is true, HMOs could provide a mechanism for selection and enrichment of *Bifidobacterium* spp. in human milk as it is being produced in the mammary gland. Indeed, we found significant associations between a few HMOs or HMO types and the presence or relative abundance of *Bifidobacterium* in milk, suggesting that certain HMO profiles could perhaps generate a favorable niche for this bacterium in milk by providing a preferential substrate for specific *Bifidobacterium* species. However, this hypothesis requires further investigation since *Bifidobacterium* and HMO metabolism pathways were not detected in a small-scale shotgun metagenomic study of the milk microbiota (16), and to our knowledge, there is currently no evidence that bacteria actively metabolize HMOs in the mammary gland.

We also provide evidence for a potential association between milk fatty acids and microbiota. The majority of studies examining fatty acids and microbial communities have focused on microbial production of short chain fatty acids in the gastrointestinal tract (55), whereas we explored associations between medium and long chain fatty acids and microbiota in milk. We observed a significant association of the fatty acids profile with the overall microbiota composition. However, this pattern emerged only when examining the summary metric of fatty acids profile (i.e., PC1) and controlling for HMO profile and confounding variables, potentially indicating a subtle impact of the fatty acids on microbiota. We also identified a few specific fatty acids to be associated with microbiota α diversity or overall composition. Stearic acid (18:0) was the fourth most abundant fatty acid in our study and was significantly associated with the overall composition of the milk microbiota independently of confounding factors.

The causative mechanism for the associations between milk fatty acids and milk microbiota is unclear since some fatty acids have antibacterial properties (20, 21) while others are produced or consumed by the microbial community. Pathways are described for recognition and uptake of exogenous fatty acids by bacteria. Internalized fatty acids will be metabolized and utilized as transcriptional regulators, source of energy, or membrane phospholipids (56). Moreover, some fatty acids are considered as potential prebiotics (57). Even though we did not observe many correlations of milk fatty acids with milk microbiota, it is possible that fatty acids alter bacterial physiology and virulence (58–61) rather than growth kinetics. Alternatively, milk fatty acids could modulate milk immune cell concentrations (13) and hence indirectly affect milk microbiota. Generally, it is unclear if milk fatty acids, which are largely bound to glycerol, are readily accessible to milk bacteria. It is possible that some fatty acids are released in the infant oral cavity by lingual lipases (62) and thus either locally affect the oral bacteria before retrograde inoculation of the mammary gland, or alternatively get transferred to the intramammary region with the retrograde milk flow during breastfeeding.

### Strengths and Limitations

The main strength of this study is our integrative, multi-variable approach to assessing the associations between HMOs, fatty acids, and milk microbiota, using the rich data and large sample size afforded by the CHILD cohort. In this study, we were able to control for maternal intake of fish oil supplement and secretor status as key factors influencing fatty acids and HMOs, respectively. Additionally, informed by our previous analysis (4), we controlled for other factors that may influence the microbiota. A limitation of our study is that milk samples were pooled from multiple feeds and not collected aseptically, in order to reflect the “average” composition of milk fed to each infant. Thus, diurnal variability and differences in foremilk and hindmilk could not be studied. Information on mastitis, which could be associated with shifts in milk microbiota and fatty acid compositions (63, 64), was not available in our study, and absolute concentrations of fatty acids were not available. Other milk components such as antimicrobial peptides and antibodies, which could confound the association of HMOs and fatty acids with milk microbiota, were not available for our samples. Finally, as with all culture-independent microbiota studies, we could not quantify bacterial load nor confirm the viability of bacteria identified in our samples (65).

## Conclusion and Future Direction

Overall, using multiple approaches for data integration to analyse three major components of human milk (fatty acids, microbiota, and HMOs), we identified strong correlations within each group, but relatively few correlations between groups. Fatty acid composition, more so than HMO profile, was associated with milk microbiota composition when controlling for relevant confounders. While the cumulative effect of HMOs on the entire microbial community appears to be small, our findings suggest that individual HMOs might promote or inhibit growth of specific milk bacteria, potentially providing a selection mechanism for vertical mother-offspring transmission of microbiota. Further research is warranted to explore these possibilities, and to determine how these and other milk components collectively modulate the infant gut microbiota and influence the risk of developing microbiota-mediated childhood diseases.

## Data Availability

The datasets generated for this study can be found in Sequence Read Archive, SRP153543.

## Ethics Statement

All participants gave written informed consent in accordance with the Declaration of Helsinki. The protocol was approved by the Human Research Ethics Boards at McMaster University, the Hospital for Sick Children, and the Universities of Manitoba, Alberta, and British Columbia.

## Author Contributions

SM and MA contributed to conceptualization. SM, EK, CF, and LB contributed to methodology. SM, FA and, KM contributed to investigation. SM, CF, LB, and MA contributed to writing the original draft. FA, KM, SS, BR, QD, AB, PM, ST, TM, DL, MS, PS, and EK contributed to writing the review and to editing. MA contributed to funding acquisition and supervision. AB, PM, ST, DL, and MS contributed to PS resources.

### Conflict of Interest Statement

The authors declare that the research was conducted in the absence of any commercial or financial relationships that could be construed as a potential conflict of interest.

## Abbreviations

HMO: human milk oligosaccharide
MUFA: monounsaturated fatty acid
PUFA: polyunsaturated fatty acid
SFA: saturated fatty acid
2′FL: 2′-fucosyllactose
3FL: 3-fucosyllactose
3′SL: 3′-sialyllactose
6′SL: 6′-sialyllactose
LNT: Lacto-N-tetraose
LNnT: Lacto-N-neotetraose
LNFP: Lacto-N-fucopentaose
LST: Sialyl-LNT
DFLNT: Difucosyl-LNT
DSLNT: Disialyl-LNT
FLNH: Fucosyl-lacto-N-hexaose
DFLNH: Difucosyl-lacto-N-hexaose
FDSLNH: Fucosyl-disialyl-lacto-N-hexaose
DSLNH: Disialyl-lacto-N-hexaose
DFLac: Difucosyllactose
LNH: Lacto-N-hexaose
TVA: Trans vaccenic acid
LA: Linoleic acid
GLA: γ-linoleic acid
ALA: α-linolenic acid
DHGLA: Dihomo-γ-linolenic acid
AA: Arachidonic acid
CLA: Conjugated linoleic acid
EPA: Eicosapentanoic acid
DHA: docosahexaenoic acid
DPA: docosapentaenoic acid.
