# Supplementary material for: Integrated Analysis of Human Milk Microbiota With Oligosaccharides and Fatty Acids in the CHILD Cohort
Source: Front Nutr. 2019 May 16;6:58. doi: 10.3389/fnut.2019.00058 (PMC6532658; doi:10.3389/fnut.2019.00058)

Supplementary Material for "Integrated Analysis of Human Milk Microbiota with Oligosaccharides and Fatty Acids in the CHILD Cohort" (Moossavi et. al.)

Supplementary Methods

Milk microbiota analysis

Microbial data pre-processing and reagent contaminant removal

Supplementary references

Supplementary Tables & Figures

Table S1. Human milk oligosaccharides (HMOs) and fatty acids structural categories

Table S2. Characteristics of mother-infant dyads from the CHILD cohort included in this analysis (n=393) in comparison with all eligible dyads (n=2536).

Figure S1. Association of maternal and infant secretor status on the overall composition of A) milk microbiota, B) HMOs, and C) fatty acids.

Figure S2. Association of milk fatty acids with milk microbiota richness (observed ASVs) and diversity (inverse Simpson index).

Figure S3. Association of milk microbiota diversity and taxonomic clusters with HMOs and fatty acids composition

Figure S4. Significant correlation of milk microbiota structure with HMOs stratified by maternal secretor status

Milk microbiota analysis

Genomic DNA was extracted from 1 ml breastmilk using Quick-DNA Fungal/Bacterial extraction kit following the manufacturer's instructions (Zymo Research, USA). The samples were centrifuged (13,000 g at 4°C for 20 min), the fat rim was carefully removed using a sterile swab, and the supernatant stored for future analysis. Total DNA was extracted from the pellet. Samples were sequenced following amplification of V4 hypervariable region of the 16S rRNA gene with modified F515/R806 primers (1) on a MiSeq platform (Illumina, San Diego, CA, USA) as previously described (2). Sterile DNA-free water was used as negative controls in sequencing library preparation. A mock community consisting of DNA extracted from of 10 species with known theoretical relative abundances (Zymo Research, USA) were also run as positive control. Overlapping paired-end reads were processed with dada2 pipeline (3) using the open-source software QIIME 2 v.2018.6 (https://qiime2.org)(4). Unique amplicon sequence variants (ASVs) were assigned a taxonomy and aligned to the 2013 release of the Greengenes reference database at 99% sequence similarity (5). Demultiplexed sequencing data was deposited into the Sequence Read Archive (SRA) of NCBI and can be accessed via accession number SRP153543.

Microbial data pre-processing and reagent contaminant removal

Data analysis was conducted in R (6). Initial preprocessing of the ASV table was conducted using the Phyloseq package (7). Potential reagent contaminants (8) were identified using decontam package based on either the frequency of the ASV in the negative control or the negative correlation with DNA concentration (9). Decontam package could remove 70-90% of contaminants specifically when the source of contamination was not well-defined (10). Overall, 9,711 unique ASVs were detected and 173 were identified as contaminants and excluded. Mock community composition was assessed and agreement with theoretical composition verified. Samples with less than 25,000 sequencing reads were excluded (n=35) and the remaining samples (n=393) were rarefied to the minimum 25,000 sequencing reads per sample. ASVs only present in the mock community or negative controls (n=894) and ASVs belonging to phylum Cyanobacteria, family of mitochondria, and class of chloroplast (n=240) were removed. ASVs with less than 20 reads across the entire dataset (n=6173) were also removed, resulting in 1972 remaining ASVs. The contribution of the excluded rare ASVs to the total reads per sample was deemed negligible. The numbers of sequencing reads of taxa were then relativized to the total sum of 25,000. This dataset was used for analysis unless otherwise specified.

We obtained an average (SD) of 47,710 (18,643) high-quality sequencing reads per sample, compared with 46,770 (13,479) reads from the mock community, and 627 (1,034) reads in negative controls. Profiles were significantly different by sample type (samples, mock community, or negative controls, $p < 0.001$) but not sequencing runs or PCR reactions. Sequencing contaminants (n=173 from 9884 total amplicon sequence variants (ASVs)) were identified and removed using the *decontam* package in R (9). Sequencing depth was not significantly impacted following contaminant removal in the samples. Additionally, the overall structure of the microbiome at higher taxonomic levels did not change in samples after contaminant removal. Overall, we observed strong consistency between the observed and expected composition of the mock community.

Supplementary References

1. Caporaso JG, Lauber CL, Walters WA, Berg-Lyons D, Huntley J, Fierer N, et al. Ultra-high-throughput microbial community analysis on the Illumina HiSeq and MiSeq platforms. *ISME J* (2012) 6(8):1621-4.
2. Derakhshani H, Tun HM, Khafipour E. An extended single-index multiplexed 16S rRNA sequencing for microbial community analysis on MiSeq illumina platforms. *J Basic Microbiol* (2016) 56(3):321-6.
3. Callahan BJ, McMurdie PJ, Rosen MJ, Han AW, Johnson AJ, Holmes SP. DADA2: High-resolution sample inference from Illumina amplicon data. *Nat Methods* (2016) 13(7):581-3.
4. Caporaso JG, Kuczynski J, Stombaugh J, Bittinger K, Bushman FD, Costello EK, et al. QIIME allows analysis of high-throughput community sequencing data. *Nat Methods* (2010) 7(5):335-6.
5. DeSantis TZ, Hugenholtz P, Larsen N, Rojas M, Brodie EL, Keller K, et al. Greengenes, a chimera-checked 16S rRNA gene database and workbench compatible with ARB. *Appl Environ Microbiol* (2006) 72(7):5069-72.
6. R Core Team. R: A language and environment for statistical computing. R Foundation for Statistical Computing, Vienna, Austria. (2017).
7. McMurdie PJ, Holmes S. phyloseq: an R package for reproducible interactive analysis and graphics of microbiome census data. *PLoS One* (2013) 8(4):e61217.
8. Salter SJ, Cox MJ, Turek EM, Calus ST, Cookson WO, Moffatt MF, et al. Reagent and laboratory contamination can critically impact sequence-based microbiome analyses. *BMC Biol* (2014) 12:87.
9. Davis NM, Proctor D, Holmes SP, Relman DA, Callahan BJ. Simple statistical identification and removal of contaminant sequences in marker-gene and metagenomics data. *Microbiome* (2018) 6(1):226.
10. Karstens L, Asquith M, Davin S, Fair D, Gregory WT, Wolfe AJ, et al. Controlling for contaminants in low biomass 16S rRNA gene sequencing experiments. *bioRxiv* (2018) 329854:doi: https://doi.org/10.1101/329854.

Table S1. Human milk oligosaccharides (HMOs) and fatty acids structural categories

Category	Components
HMOs	
Small HMOs	2’FL, 3FL, 3’SL, 6’SL
Type 1 (Terminal galactose attached in β1-3 linkage)	LNT, LNFP I, LNFP II, LSTb, DSLNT
Type 2 (Terminal galactose attached in β1-4 linkage)	LNnT, LNFP III, LSTc
α-1,2	LNFP I, 2’FL
α-1,3	LNFP III, 3FL
α-2,6	LSTb, LSTc, 6’SL
Fucosylated/Sialyated	FDSLNH
Fucosylated/Non-Sialyated	2’FL, 3FL, DFLac, LNFPI, LNFPII, LNFPIII, DFLNT, FLNH, DFLNH
Non-Fucosylated/Sialyated	3’SL, 6’SL, LSTb, LSTc, DSLNT, DSLNH
Non-Fucosylated/Non-Sialyated	LNnT, LNT, LNH
Fatty acids	
Saturated Fatty Acids (SFA)	10:0, 12:0, 14:0, 15:0, 16:0, 17:0, 18:0, 20:0, 24:0
Monounsaturated Fatty Acids (MUFA)	14:1n9, 16:1n9, 18:1n9, 24:1n9
Polyunsaturated Fatty Acids (PUFA)<br>n-3<br>n-6	 18:3n3, 20:4n3, 20:5n3, 22:5n3, 22:6n3,<br>18:2n6, 18:3n6, 20:2n6, 20:3n6, 20:4n6, 22:5n6, 22:4n6
De novo synthesis	10:0, 12:0, 14:0
Diet, liver synthesis, or body storage	15:0, 16:0, 17:0, 18:0, 20:0, 24:0<br>14:1n9, 16:1n9, 18:1n9, 24:1n9<br>18:3n3, 20:4n3, 20:5n3, 22:5n3, 22:6n3,<br>18:2n6, 18:3n6, 20:2n6, 20:3n6, 20:4n6, 22:5n6, 22:4n6

Table S2. Characteristics of mother-infant dyads from the CHILD cohort included in this analysis (n=393) in comparison with all eligible dyads (n=2536).

Factor	Mean ± SD or n (%)[a]	
	Subset for this study N=393	All eligible dyads N=2536[b]
**Maternal factors**		
Age (years)	33.0±4.2	32.7±4.2
Pre-pregnancy BMI (Kg/m^2)	24.3±5.2	24.5 ± 6.3
Secretor status [c]	279 (71.7)	-
Ethnicity		
Caucasian	287 (73.0)	1870 (74.3)
Asian	73 (18.6)	403 (16.0)
First Nations	15 (3.8)	87 (3.5)
Other	18 (4.6)	158 (6.3)
Fish oil supplement use	81 (20.8)	570 (23.4)
**Infant factors**		
Birth weight (g)	3469±469	3450±479
Female sex	192 (48.3)	1198 (47.2)
Gestational age (weeks)	39.2±1.3	39.2±1.2
Secretor status	289 (78.7)	1718 (78.5)
**Early life factors**		
Mode of delivery		
Elective C/S	46 (11.9)	264 (10.6)
Emergency C/S	47 (12.1)	339 (13.6)
Vaginal	294 (76.0)	1893 (75.8)
**Breastfeeding**		
Lactation stage at sample collection (weeks)	17.3±5.3	16.5±5.0
Some indirect BF (at least one serving of pumped milk in the past two weeks)	226 (58.1)	1518 (59.9)
**Milk components**		
Total HMO concentration (mg/mL) [c]	10.2±2.1	-
Saturated fatty acids (%) [c]	40.9±5.0	-
Monounsaturated fatty acids (%) [c]	39.3±3.5	-
Polyunsaturated fatty acids (%) [c]	16.7±3.7	-

[a] Percentages are calculated after excluding dyads with missing data. BF, breastfeeding; BMI, body mass index; C/S, caesarean section; HMO, human milk oligosaccharide;
[b] Those who breastfed ≥12 weeks and provided a milk sample
[c] HMO and fatty acid data are only available among those selected for milk composition analysis.

Figure S1. Association of maternal and infant secretor status on the overall composition of A) milk microbiota, B) human milk oligosaccharides (HMOs), and C) fatty acids.

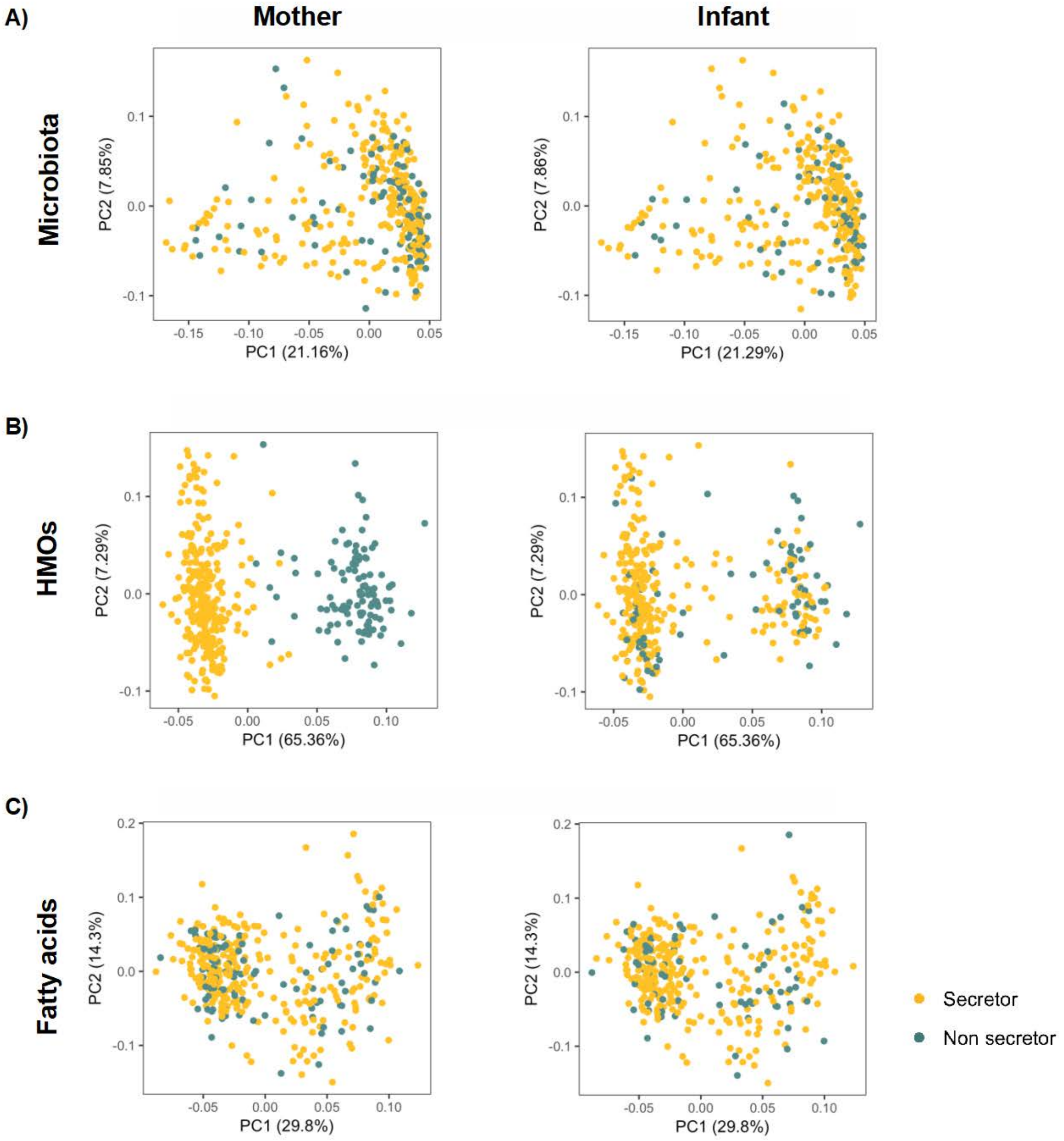

Figure S2. Association of milk fatty acids with milk microbiota richness (observed ASVs) and diversity (inverse Simpson index). Adjusted for mode of breastfeeding, infant sex, parity, and birth mode.

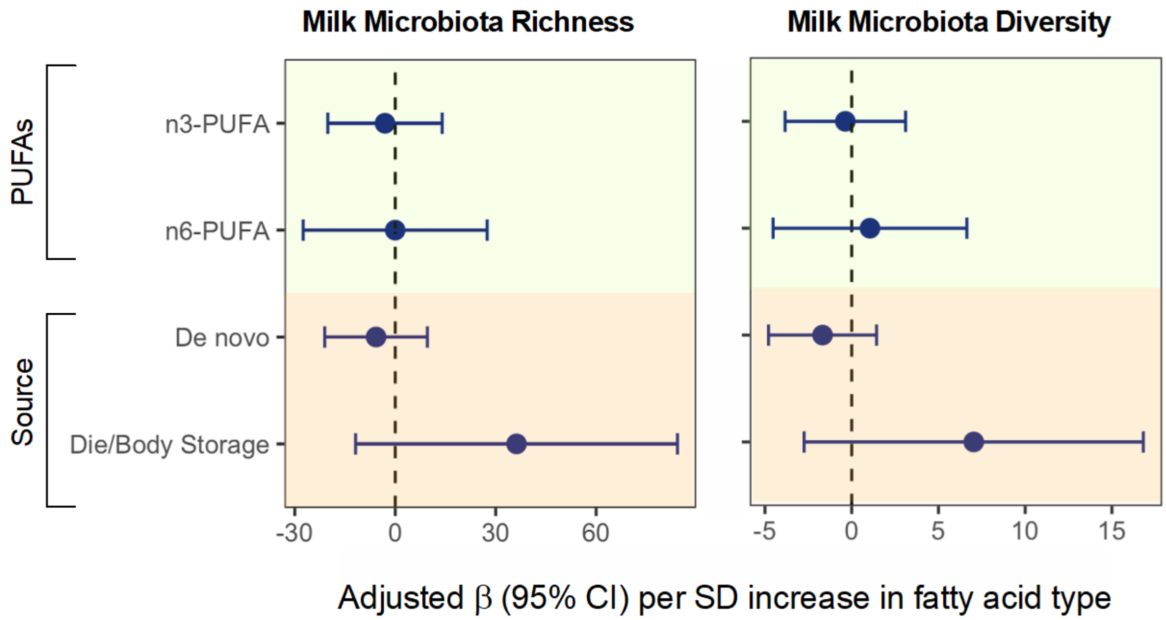

Figure S3. Association of milk microbiota diversity and taxonomic clusters with human milk oligosaccharides (HMOs) and fatty acids composition

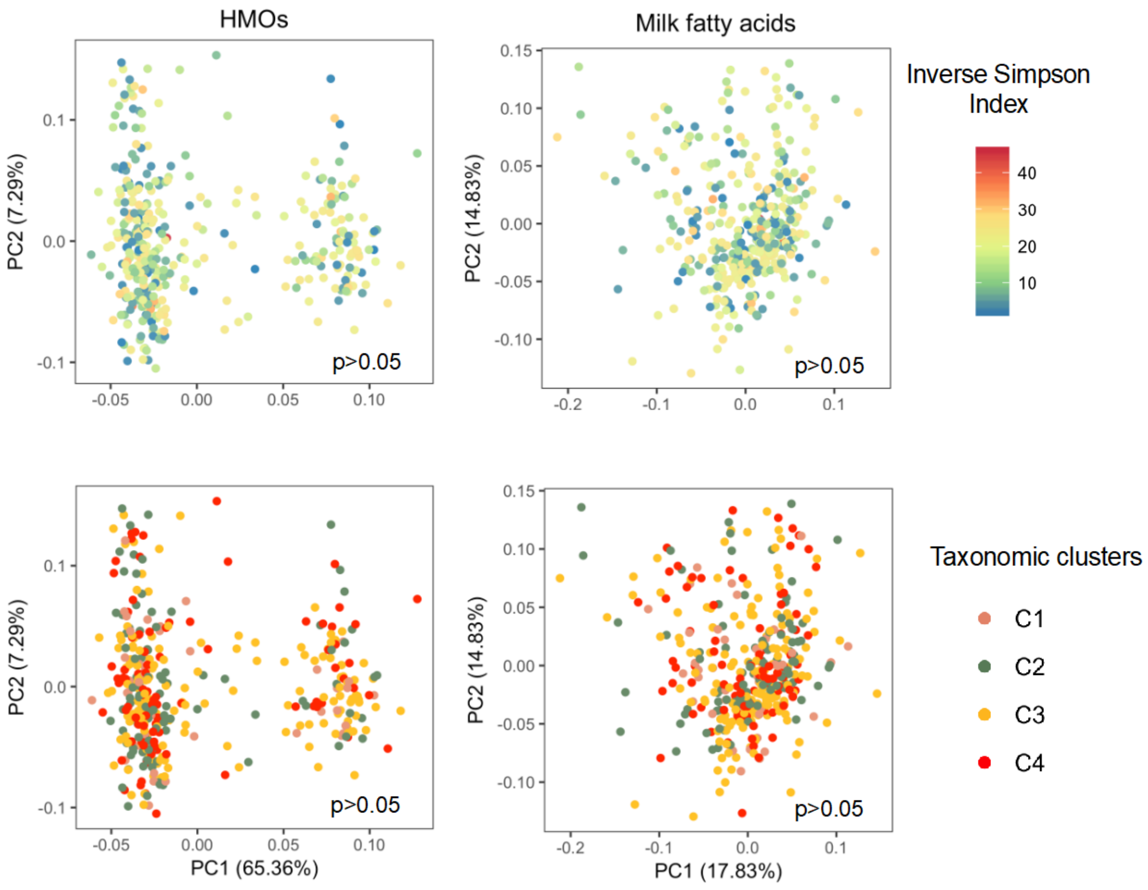

Figure S4. Significant correlation of milk microbiota structure with human milk oligosaccharides (HMOs) stratified by maternal secretor status

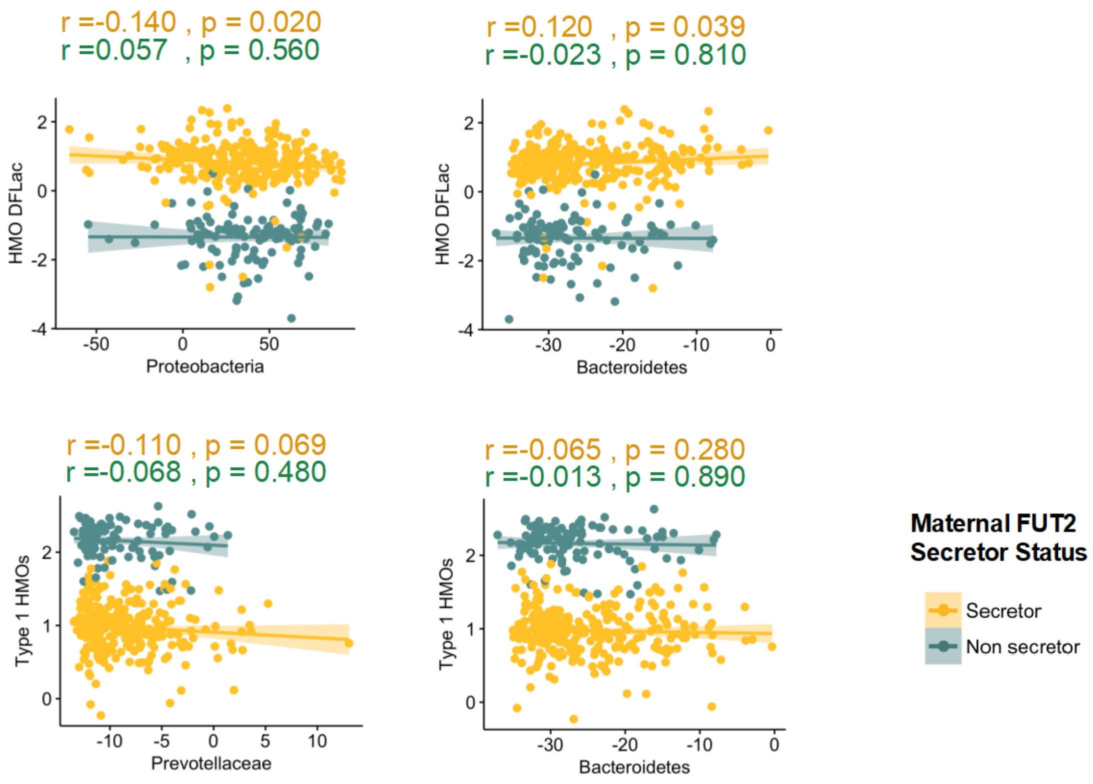

Supplement: Supplementary file 1 [file Data_Sheet_1.pdf]
